# Highly Invasive Biliary Cancer Presenting as Multiple Extrabiliary Lesions Masquerading as Abscesses

**DOI:** 10.7759/cureus.48894

**Published:** 2023-11-16

**Authors:** Alexander Lu, Alireza Mojtahedi, Lukman Cheraghvandi, David Wynne, Neda Zarrin-Khameh, Mohammad Ghasemi Rad

**Affiliations:** 1 Department of Radiology, Baylor College of Medicine, Houston, USA; 2 Department of Pathology & Immunology, Baylor College of Medicine, Houston, USA

**Keywords:** bile duct cancer, metastatic gall bladder, endoscopic ultrasound (eus), biliary carcinosarcoma, carcinosarcoma

## Abstract

Carcinosarcomas of the biliary tract are an extremely rare type of malignancy and may be low on a differential when presenting as multiple metastatic masses. In this case report, we report a case of a female who presented with an aggressive late-stage disease whose initial workup did not indicate a malignant process. Further complicating her care, biopsy samples taken from extra-hepatic masses were culture-positive for *Lactobacillus rhamnosu*. Given the late stage of the patient’s disease, hospice care was initiated. The patient passed away four months after the initial presentation.

## Introduction

Carcinosarcomas of the bile duct are a relatively rare form of tumor appearing in the hepatobiliary system. Histologically, these tumors are characterized by both mesenchymal and epithelial tissues [1]. To date, fewer than 100 cases have been reported in the literature since its initial description in 1097 [2]. Here, we report a case of a biliary carcinosarcoma and its presentation, imaging, histology, and clinical management to further inform the management of this rare tumor.

## Case presentation

**Figure 1:**
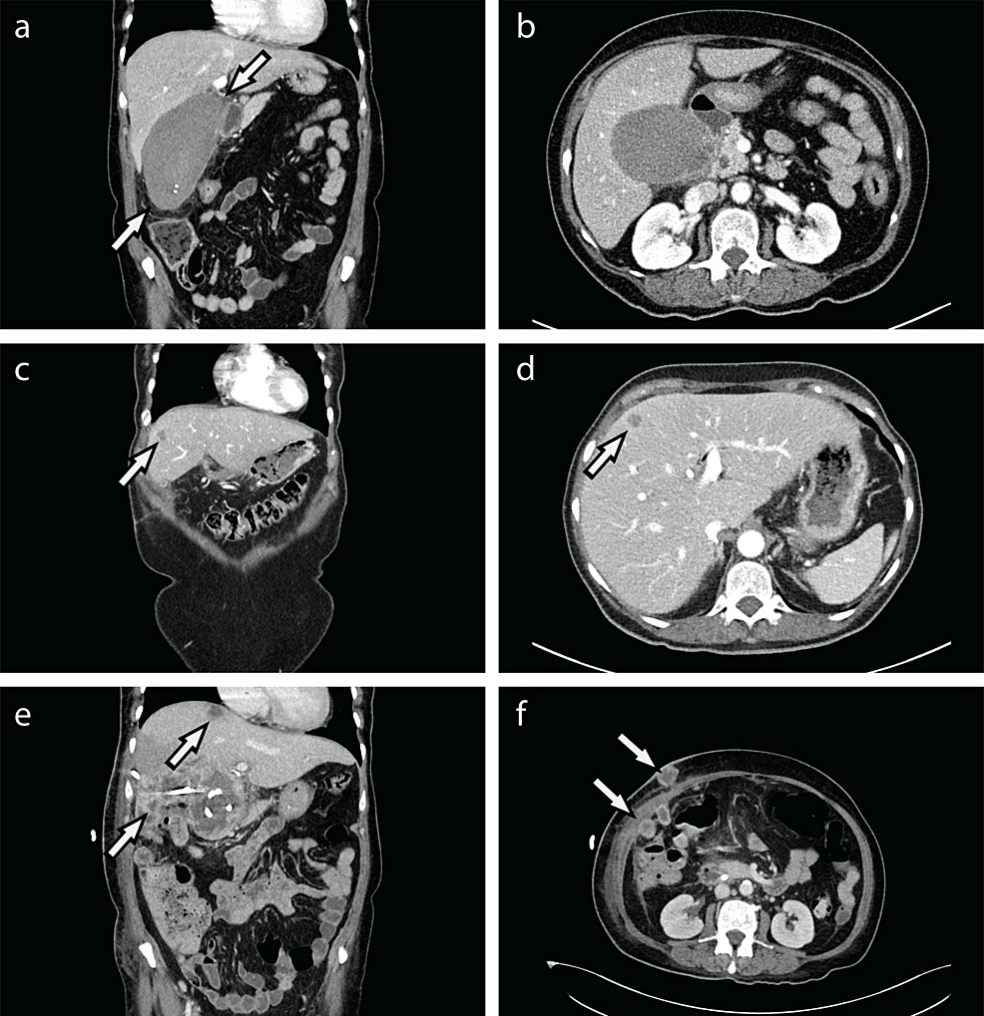
CT images upon initial admission and post-drainage (a/b) Coronal and axial CT imaging with contrast of the abdomen upon initial admission to ED. The length of the gallbladder measured along its greatest length was approximately 13 cm (between the white arrows). (c/d) Coronal and axial CT imaging with contrast of the abdomen upon readmission to ED. There are multiple new hypoattenuating lesions in the liver (white arrows). No lesions of the omentum or abdominal wall are noted. (e/f) Repeat CT imaging following three rounds of CT-guided drainage. There are now multiple lesions involving the liver, omentum, and anterior abdominal wall containing internal fluid components with peripherally thick, irregular, enhancing walls.

A 65-year-old female with a past history of type 2 diabetes mellitus (T2DM), hypertension, and systemic lupus erythematosus (SLE) presented to the emergency department (ED) with a six-day history of right upper quadrant (RUQ) pain associated with nausea, vomiting, and chills. Point-of-care ultrasound of the RUQ showed distention in the gallbladder with heterogeneous shadowing and non-shadowing hypoechoic material. Computed tomography (CT) imaging of the abdomen revealed a grossly distended gallbladder with focal irregular thickening of the gallbladder neck (Figures 1a-1b). Based on these findings, the patient was then admitted for operative management of presumptive acute cholecystitis. She underwent an uncomplicated open cholecystectomy with drain placement in the gallbladder fossa. The specimen retrieved was sent for pathology and reported as acute cholecystitis negative for malignancy, in line with the preoperative diagnosis. The following day, an endoscopic retrograde cholangiopancreatography (ERCP) was done due to concern for intra-operative bile leakage, and a common bile duct (CBD) stent and sphincterotomy were performed. At discharge, she was stable with appropriate follow-up care. 

One month later, the patient presented again to the ED with several weeks of worsening abdominal pain. CT imaging showed a 4.9 cm abscess in the gallbladder fossa, with multiple new hypoattenuating lesions in the liver that were suggestive of micro-abscesses (Figures 1c-1d). CT-guided aspiration with drainage catheter placement of the gallbladder fossa yielded 10 mL of brown/red thick fluid. CT imaging repeated two days later showed further growth of the lesions. Drainage was repeated four days later due to continued enlargement of the lesions, which were causing a mass effect on surrounding structures. Two days after the repeat drainage, imaging showed improvement in gallbladder fossa lesion size but continued enlargement of hepatic abscesses. At this time, additional lesions were also noted in the peritoneum and along the laparotomy scar. A decision was then made to perform CT-guided drainage for a third time for intrahepatic lesions, which yielded 9 mL of thick serosanguinous fluid. An ERCP with endoscopic ultrasound (EUS) was then performed to swap out the previously placed CBD stent. Two fine-needle biopsies of a 55 mm heterogenous hypoechoic mass were also taken under EUS. Two days following ERCP, repeat CT abdomen and pelvis showed continued growth of multiple lesions involving the liver, omentum, and anterior abdominal wall concerning for superimposed infection or neoplastic process (Figures 1e-1f). Given these new findings, exploratory surgery was done, which allowed for the visualization of multiple hepatic and gallbladder fossa lesions. Lesions were incised using cautery and the caseous necrotic contents were evacuated via suction. There were no complications intra- or postoperatively. The final pathology report indicated high-grade sarcoma. The patient and family subsequently declined further treatment and opted for hospice care. She passed away approximately four months after her initial ED admission.

**Figure 2:**
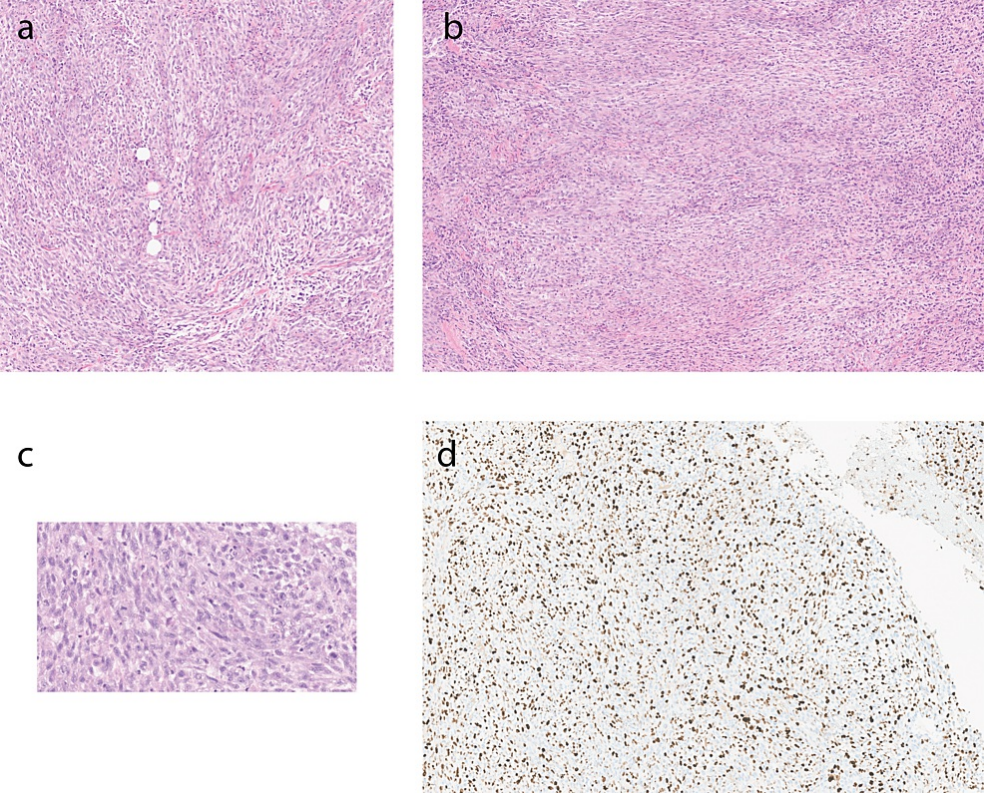
Carcinosarcoma with hematoxylin and eosin and Ki-67 stains (a) Tumor cells show round spindled cells in myxoid and loosely fascicular pattern with atypia. There is mild acute inflammatory infiltrate including eosinophils. Note a few mature adipocytes in the center. (b) Tumor cells show round spindled cells in myxoid and loosely fascicular pattern with atypia. (c) Tumor cells show marked atypia. Few mitotic figures can be identified (hematoxylin-eosin, original magnification X40). (d) Ki-67 proliferation marker showing marked increased nuclear staining in tumor cells with a high proliferation index of 65% ((a-c) Hematoxylin-eosin, (a,b) original magnification X10, (c) original magnification 40x).

Histologically, the tumor was characterized as a high-grade malignant mesenchymal tumor with storiform and fascicular patterns arrangement of highly atypical spindled and polygonal cells as well as frequent foci of coagulative necrosis (Figure 2). Immunohistochemical (IHC) staining demonstrated focally positive staining for SMA and pancytokeratin, and negative staining for ALK1, CD10, S100, SOX10, desmin, WT-1, ER, PAX-8, CD34, inhibin, and EMA. These findings were consistent for three different samples taken from an intra-abdominal abscess, abdominal wall plaque, and liver abscess during the exploratory laparotomy.

## Discussion

Carcinosarcoma in the hepatobiliary system is an exceedingly rare tumor, and as such, may be low on the differential for metastatic abdominal masses. Frustratingly, a preoperative diagnosis is difficult with serum markers such as CEA and CA 19-9 fluctuating between reported cases [2,3]. Imaging, likewise, does not provide clarity, as hypoattenuating lesions with peripherally thick irregular enhancing walls is a nonspecific finding [4,5]. Preoperative biopsy under EUS or CT imaging may be warranted for rapidly expanding lesions, although the utility of the preoperative diagnosis has not yet been established due to the rarity and lack of consensus on treatment. In general, it appears that the current standard is surgical resection [6]. Definitive diagnosis and treatment of the patient were delayed due to several factors, including a low index of suspicion, lack of diagnostic tests, as well as confounding positive cultures for *Lactobacillus rhamnosus* from the wound and drained abscess contents.

It has been noted that carcinosarcomas are more locally aggressive and may metastasize earlier in the disease process [2]. In the above patient, the time between the initial imaging and the first set of images with notable masses was less than one month. As the patient in this case declined further treatment following surgical resection, it is difficult to prognosticate long-term outcomes. However, the prognosis of this disease in general is poor. Cases as described in literature are almost universally fatal, even after surgical resection and adjuvant therapy. Aldossary et al. reported a single case with a recurrence-free survival time of up to 86 months at the time of publication [7]. However, Zhang et al. reported a mean survival time of 17.5 months in their meta-analysis, making 86 months of disease-free survival a likely outlier [8]. Other meta-analyses have shown even shorter survival times and have also noted that demographic factors are an independent variable for survival [9]. For a more accurate comparison, we have compiled all cases of biliary sarcoma with confirmed Union for International Cancer Control (UICC) Stage IVB disease since 1970 that we were able to find (Table 1).

**Table 1:** Comparison between UICC Stage IVb disease patients since 1970 UICC: Union for International Cancer Control

	Our Patient 2023	Aldossary (2019) [7]	Aldossary (2019) [7]
Age/Gender	65/F	40/M	52/F
Survival Time	4 months	6 months	3 months
Treatment Method	Surgical Resection	Surgical Resection	Surgical Resection
Adjuvant Treatment	No	Yes, chemotherapy	No

Given the relatively indolent progression of the disease, many patients only present for assessment when the disease is locally advanced. At this time, it is still undermined what roles chemo- or radiotherapy may have in management. Further work needs to be done but given the rare nature of this pathology, head-to-head studies are unlikely. As previously mentioned, serum molecular markers have been unreliable for diagnosis, but several genetic abnormalities have been identified that may provide targets for molecular therapy for related gall bladder cancers. Loss of activity of TP53 and overactivation of COX2 have been implicated in carcinogenesis in gall bladder cancers and may provide appropriate targets for advanced therapies.

## Conclusions

In conclusion, we present a case of carcinosarcoma of the hepatobiliary system that presented with multiple extra-hepatic lesions. This previously unreported presentation had features such as positive cultures from the fluid drained from the lesions, which delayed appropriate diagnosis, but, ultimately did not change the management of the patient. Given the rare nature of this malignancy, more study is required to identify at-risk patients and optimal treatment modalities.

Informed consent from next of kin was not required due to institutional policies on the publication of de-identified patient information. Official certification of de-identification was obtained prior to publication.
